# Perceived influence of value systems on the uptake of voluntary medical male circumcision among men in Kweneng East, Botswana

**DOI:** 10.1080/17290376.2020.1810748

**Published:** 2020-11-17

**Authors:** Thandisizwe R. Mavundla, Fungai Mbengo, Khanyenda Bruce Ngomi

**Affiliations:** a Department of Health Studies, College of Human Sciences, University of South Africa, Pretoria, South Africa; b School of Nursing and Midwifery, Edith Cowan University, Joondalup, Australia

**Keywords:** Botswana, Culturally congruent, Perceptions, Value systems, Voluntary medical male circumcision

## Abstract

Botswana is one of the countries in Eastern and Southern Africa significantly impacted by the Human Immunodeficiency Virus (HIV). To control the spread of HIV, the government in 2009 rolled out the voluntary medical male circumcision (VMMC) programme as an additional HIV prevention strategy with the goal of circumcising 80% of HIV negative men by 2016. However, the country failed to achieve this goal as less than 30% of the targeted men were circumcised by 2016. A study was therefore conducted to explore and describe the factors that are perceived by men in Botswana to influence the uptake of VMMC in order to inform future policymaking and programming on VMMC. An exploratory descriptive, qualitative design was utilised to investigate perceived factors influencing the uptake of VMMC among men. Data were collected from 38 men, aged 18–49 years in Kweneng East, Botswana using semi-structured individual interviews and focus group discussions (FGDs). Tesch's method of qualitative data analysis was used to code and categorise transcribed data into meaningful themes. Upon analysis, three themes emerged as influencing the uptake of VMMC: (a) the influence of value systems associated with stakeholder consultation in the community; (b) the influence of value systems associated with cultural beliefs and (c) the influence of value systems associated with religious beliefs. The influence of value systems associated with stakeholder consultation in the community was found to manifest in the form of the lack of consultation with men at the inception of the VMMC; the lack of involvement of village elders during the service delivery process and the lack of involvement of women in VMMC. In addition, the influence of value systems associated with cultural beliefs was found to manifest in the form of the lack of openness between parents and children on sexual matters and the lack of traditional leadership support in VMMC. Lastly, the influence of value systems associated with religious beliefs was found to manifest in the form of religious views not in support of the VMMC and religious views in support of the VMMC. It is concluded that value systems associated with stakeholder consultation, cultural beliefs and religious beliefs were the factors influencing the uptake of VMMC among men in Kweneng East, Botswana, and these factors to a larger extent deterred men from using VMMC services. Based on these findings, it is therefore concluded that government and other providers of VMMC should consider the influence of value systems on the uptake of VMMC in order to provide culturally congruent VMMC services and boost of the uptake of VMMC among men in Kweneng East, Botswana.

## Introduction

Human immune deficiency virus is an important public health challenge worldwide. However, poor countries such as those in Eastern and Southern Africa are disproportionately affected by the HIV epidemic. Of the estimated 37.9 million people living with HIV worldwide, approximately 20.6 million people (54%) are from Eastern and Southern Africa (Joint United Nations Programme on HIV/AIDS, 2019). Evidence indicates that most of the HIV infections in this region are acquired through heterosexual contact (Idele et al., 2014).

Botswana is one of the countries in Eastern and Southern Africa significantly impacted by HIV. Botswana is home to an estimated 2.3 million people, and of these 384,000 (17%) are living with HIV (Joint United Nations Programme on HIV/AIDS, 2019; Worldometers, 2019). Moreover, the latest national survey carried out in 2013, estimated the national HIV prevalence was 18.5% compared to 17.6% observed in 2008 among the population aged 18 months and above (Statistics Botswana, 2014). HIV prevalence differed by district and ranged from 11.1% in Kgalagadi South to the highest of 27.5% in Selebi-Phikwe (Statistics Botswana, 2014).

HIV has negative outcomes not only on the infected individuals but also on children, families and society (Boutayeb, 2009; Ji, Li, Lin, & Sun, 2007; Lozano et al., 2012). In Botswana, it has been estimated that the HIV epidemic costs taxpayers approximately US$159 million per annum (Joint United Nations Programme on HIV/AIDS, 2019).

In response to the prevalence public health challenge of HIV, the government implemented a series preventive interventions before 2009, which included the prevention of mother-to-child transmission (PMTCT), prevention education, antiretroviral therapy, and routine HIV testing and counselling (Ministry of Health, 2009). However, the prevalence of HIV in Botswana remained one of the highest with a prevalence of 17.6% in the general population in 2008 (Central Statistics Office, 2009).

Also, traditional initiation for males into manhood (done for a group of men at the same time to assess their collective masculine strength) in Botswana, generally involved undergoing circumcision (Meissner & Buso, 2007; Douglas et al., 2012). However, the use of one knife to circumcise all male initiates which was considered as indecent and barbaric by the then missionaries and therefore enforced its abolishment during the twentieth century (Comaroff, 1985) is still upheld in this modern times by tribes such as the Mochudi who have revived it and see it as an identity of their tribe (Setlhabi, 2014). There are also other traditionally non-circumcising communities such as the Bakwena tribe in Botswana (Mavundla & Maibvise, 2013). Moreover, there was evidence from three randomised controlled trials conducted in Kenya, South Africa and Uganda reporting that male circumcision is effective in reducing the risk of HIV transmission from infected woman to an uninfected man by approximately 60% (Auvert et al., 2005; Bailey et al., 2007; Gray et al., 2007). In the face of unsafe traditional male circumcision practices, non-circumcising practices as well as the high prevalence of HIV and evidence supporting the effectiveness of male circumcision in reducing the risk of HIV transmission, there was the need for the Ministry of Health to introduce the VMMC as an additional strategy to already existing HIV prevention efforts targeting HIV negative males aged 0–49 years with the aim of reaching 80% coverage by 2016 (Ministry of Health, 2009; Ngomi, 2014).

During the latter part of 2009, the government also launched a 6 months’ multimedia campaign that disseminated information through billboards, branding of public transport buses, television, radio and the public address system at strategic places in order to promote the uptake of VMMC (Sabone et al., 2013). In addition, there were efforts to increase the demand for VMMC through house-to-house mobilisation and social media (Joint United Nations Programme on HIV/AIDS, 2017; National AIDS Coordinating Agency, 2015). However, despite these initiatives, the country failed to achieve its VMMC goal; as less than 30% of the targeted men were circumcised by 2016 (Joint United Nations Programme on HIV/AIDS, 2016, 2017). This study was therefore conducted to explore and describe the factors that are perceived by men in Botswana as influencing the uptake of VMMC in order to inform future policymaking and programming on VMMC. To our knowledge, there is limited research conducted to explore men's perceptions regarding the factors influencing the uptake of VMMC among men in the context of Botswana. The study was guided by the theory of culturally congruent care which stipulates that clients’ culture, beliefs and value systems should be considered when developing and implementing health care interventions (Abitz, 2016; Albougami, Pounds, & Alotaibi, 2016; Schim, Doorenbos, Benkert, & Miller, 2007).

## Materials and methods

An exploratory, descriptive qualitative design was used to investigate the perceived factors influencing the uptake of VMMC among men in Botswana. The exploratory, descriptive qualitative design was considered appropriate for the study as the study sought to explore and describe the factors that men perceive as influencing the uptake of VMMC (Polit & Beck, 2017). The study was conducted in Kweneng East district which has a population of 256,833. The HIV prevalence in Kweneng East ranged from 18.6% to 21.5% and was higher than the national HIV prevalence of 18.5% (Statistics Botswana, 2014). The district consists of several villages including Mmopane, Metsimotlhabe, Kumakwane, Molepolole and Lentsweletau (Ngomi, 2014). However, Molepolole and Lentsweletau villages were purposefully selected to recruit men for the study. The two villages are mainly inhabited by the Bakwena people, who are one of the eight major tribes in Botswana that do not practice traditional male circumcision, therefore were considered to comprise men who were knowledgeable about VMMC and would provide rich data regarding the factors influencing the uptake of VMMC (Ngomi, 2014). The study population included all men aged between 18 and –49 years either circumcised or uncircumcised. Those included in the study were of the Bakwena tribe and willing to participate in the study. Purposive sampling method was used to recruit men who were knowledgeable about VMMC. The *kgotla* (ward) leaders based in the respective villages assisted the researchers in recruiting men for the study.

To obtain a better understanding of the factors influencing VMMC among men in Botswana, both semi-structured individual interviews and focus group discussions (FGDs) were used to collect data. Focus group discussions were conducted first with both circumcised and uncircumcised men in order to obtain multiple viewpoints regarding the factors influencing the uptake of VMMC. At the end of the FGDs participants who had indicated that they were uncircumcised were selected for the individual interviews to elicit more information regarding the reasons for not using VMMC services. The individual interviews and FGDs were conducted until data saturation or until no new themes emerged (Polit & Beck, 2017). In total five FGDs were conducted, with each group comprising of at least 6 members, and each discussion lasting approximately 60 min. In addition, five individual interviews were conducted, with each interview lasting approximately 30 min. Overall, 38 men were selected for the individual interviews and FGDs.

The individual interviews and the FGDs took place in comfortable and secure venues that enabled participants to freely express their views. An interview guide was used during the individual interviews and FGDs to ensure more clarifying, probing and that no important information is left out during the interviewing process. The interview guide covered background information about participants and questions to elicit men's perceptions regarding the factors influencing the uptake VMMC. The individual interviews and FGDs were conducted by trained facilitators who understood Setswana – an indigenous language that is spoken in the country. Facilitative communication skills were employed to encourage participants to express their views. Complementary field notes were taken during the individual interviews and FGDs. The individual interviews and the FGDs were tape-recorded and later transcribed verbatim into text. The individual interviews and FGDs were conducted in Setswana, and the transcripts were coded when they were still in the vernacular language and later translated into English. Tesch’s (1990) method of qualitative data analysis cited in Creswell (2014) was used to code and categorise transcribed data into meaningful themes.

To ensure trustworthiness, two researchers coded and categorised the data into themes, and continuously cross-checked with one another for consistency of assigned themes. Member checking was done, where a summary of the major findings or themes were provided to the participants to verify if the findings or themes corresponded with their views. Triangulation was employed by collecting data using both individual interviews and FGDs to obtain a comprehensive understanding of the perceived factors influencing the uptake of VMMC among men in Botswana.

Ethical approval for the study was obtained from the Health Studies Higher Degrees Committee of the College of Human Sciences at the University of South Africa, reference number: HSHDC 61/2011. The study was also approved by the Botswana Ministry of Health, reference number: PPME-13/18/1 Vol V11(91). Further approval was granted by the Scottish Livingstone Hospital, Molepolole, Botswana, reference number: SLH 3/241(42) and later allowed by the traditional leaders of Molepolole and Lentsweletau villages. The entire study was fully explained to the prospective participants before obtaining their written informed consent. Participants signed consent forms prior to their participation in the study to document their voluntary participation into the study. Confidentiality and privacy were ensured by conducting the individual interviews and the FGDs in secured venue and not revealing any participant information that could be used to trace or track any of the study participants. Also, the collected data were locked away in a filing cabinet and the investigators were the only persons with access to the stored data.

## Results

Schwartz (2012) defines value systems as what people consider important in their lives. Therefore, the value that people attach to health services such as the VMMC influences how they accept and utilise such services. Upon analysis of data, three themes emerged showing the influence of value systems on the uptake of VMMC. The identified themes were; (a) The influence of the value systems associated with stakeholder consultation in the community; (b) The influence of the value systems associated with cultural beliefs and (c) the influence of the value system associated with religious beliefs. These themes are discussed below.

### Theme (a): The influence of value systems associated with stakeholder consultation in the community on the uptake of VMMC

Stakeholder consultation or engagement is the process of involving those who have a role or are interested in and/or are affected by the activities of a programme (Ngomi, 2014). This consultation comprises different activities such as dialogue, collaboration in identifying problems and solution, partnering in implementation and evaluation, capacity building and empowerment. Wider stakeholder consultation in programme design and implementation leads to mutual benefit that encourage people's participation and ownership of the programme (Ngomi, 2014). The theme of the influence of value systems associated with stakeholder consultation in the community is discussed according to the categories identified below.

#### Lack of consultation with men at the inception of VMMC

The study showed that consultation of men was considered important in the Bakwena culture, however, most participants lamented that the government did not consult them at the inception of the VMMC. The participants further indicated that as a result of this lack of consultation by government they were not informed and lacked information about the advantages and disadvantages of VMMC. Providing adequate information on an issue like VMMC will go a long way to influence its uptake. The following are some of the views stated by the participants demonstrating the lack of consultation with men at the inception of the VMMC:

“Many people [men] have not done it [undergone circumcision], because from the initial stages we were not consulted about the programme and told about the advantages of it.”, “ … “Many people [men] do not know the advantages […] consultation should have been done first.”, “Government authorities should have discussed the whole issue with the nation … ”, “ … only considered the dangers of the foreskin.”

#### Lack of involvement of the village elders during the service delivery process

Majority of the participants indicated that the involvement of village elders in key decision making processes such as the implementation of the VMMC was important to them, however they lamented that village elders were not involved during the implementation of the VMMC. The lack of involvement of the village elders, who could have had an influence on men to undergo male circumcision was highlighted as an important factor that deterred men from using VMMC. This was revealed by the participants, who, when asked what they thought the government authorities should have done, considering that men were not properly consulted before implementation commenced, responded as follows:

They should have started at the top [with the village elders] in the community, until it reaches the family […] because if you deal with the elders of the villages, they further go to discuss with the people in the village […] the village elders later take the information to the kgotlas [wards] […] from there the message will be able to reach the families, and finally individuals.

Even the health workers are not involving parents or even to reach them to give information so that they can support the young ones who intend to go for circumcision […] because if the health workers interact with the elders, then we shall be encouraged to do it.

#### Lack of involvement of women in VMMC programmes

The participants revealed the need for government to involve women in VMMC initiatives as women encourage men to utilise VMMC. However, they indicated that women were not involved in the delivery of the VMMC. This lack of involvement of women in VMMC programmes was stated as an important factor that hindered men from using VMMC. When the participants were asked whether women are involved and supportive of VMMC, most of the men had this to say:

Female partners should be consulted as well, as this will also benefit them […] because they play a role by encouraging us and giving moral support. But, in the final analysis, it is one's choice either to do it or not.

The good thing is that we do not think there are some female partners who discourage their men […] if there are some, then they are very few […] the government authorities did not just consult and involve people!

The right thing to do is to discuss with the female partners and arrive at an agreement. They may also end up encouraging us to go for circumcision when they understand.

### Theme (b): The influence of value systems associated with cultural beliefs on the uptake of VMMC

In addition influence of value systems associated with stakeholder consultations, this study revealed that cultural beliefs form part of the value systems of the Bakwena people and had an influence on the uptake of VMMC. The theme of the influence value systems on the uptake of VMMC is discussed within the categories mentioned below.

#### Lack of openness between parents and children on sexual matters

A salient issue that emerged from the individual interviews and FGDs was the lack of openness related to VMMC. Participants indicated that the Bakwena culture does not permit openness between parents and children on sexual matters. Due to the lack of openness between parents and children on sexual matters young men were lacking important information about VMMC from their parents so that they can voluntarily undergo VMMC. For example, when the men were asked how often discussions take place between parents and children (young people of reproductive age) on this subject, the following are some of the views that were articulated:

It is a taboo for parents to discuss sexual issues with us young people […] they don't interact with us regarding sexual issues. That is how it is.

But because of the taboos on sexual discussions we cannot share or be open with our parents.

It is a cultural norm for our parents not to discuss certain issues with us […] and it is not a sign of respect to ask elderly people about these issues.

#### Lack of traditional leadership support

Participants highlighted the support of traditional leaders is important to enhance the successful implementation of VMMC programmes. However, participants complained about difficulties related in obtaining the support of traditional leaders. When asked about how much support they receive from the traditional leadership in VMMC, these were the responses of some of the participants:

It is difficult to support us […] like we said earlier that it is a taboo […] so they cannot say much …] even if they talk about it, they just tend to limit the extent of discussion. They can't get deeper […] maybe for only a short period of time, and quickly change the subject.

### Theme (c): The influence of value systems associated with religious beliefs on the uptake of VMMC

Value systems associated with religious beliefs came out to be an important factor influencing the uptake of VMMC. The views of some of the participants showed that the religious views not in support of VMMC had a negative effect on the way men perceive VMMC in the prevention of HIV infection. Below are some of the religious beliefs against VMMC expressed by the participants.

When it comes to the Bible, circumcision is an old practice in the Old Testament. As such, going back to it will be like going back to the old practice.

The Bible teaches us not to please the flesh. Therefore, we should live as per God's expectation and stay away from earthly activities […] there is no way I can advise people to do male circumcision while I know that it is ungodly.

However, other participants held religious views that were in support of the VMMC, as they expressed appreciation at what the government is doing. These are some of the responses that were articulated:

Whether you are a believer or not, let us do what the government has asked us to do, otherwise we will perish, because the government is fighting a good cause. Some of us are alive because of ARVs [an intervention implemented by the government]. So let the government continue with this programme […] it is either believers take it or not. The government is doing things right. Take it or leave it.

It is upon an individual to choose between good and evil […] God gave us that choice.

The views expressed by the participants pertaining to religious beliefs on VMMC clearly indicate that the values embodied in religion do dictate people's perceptions on VMMC. For this reason, religion can either promote or hinder the uptake of VMMC.

## Discussion

The purpose of this study was to investigate to explore and describe the factors that are perceived by men in Botswana as influencing the uptake of VMMC in order to inform future policymaking and programming on VMMC. The findings of this study revealed that value systems associated with stakeholder consultation in the community, cultural beliefs and religious beliefs were the factors influencing the uptake of VMMC among men in Botswana. The influence of value systems associated with stakeholder consultation in the community was found to manifest in the form of the lack of consultation with men at the inception of the VMMC; the lack of involvement of village elders during the service delivery process; and the lack of involvement of women in VMMC. The influence of value systems associated with cultural beliefs was found to manifest in the form of the lack of openness between parents and children on sexual matters; and the lack of traditional leadership support. The influence of value systems associated with religious beliefs was found to manifest in the form of religious views not in support of the VMMC and religious views in support of the VMMC. These factors to a larger extent deterred men from using VMMC services.

Most participants in this study cited the lack of consultation with men at the inception of VMMC, lack of involvement of village elders during the service delivery process as barriers of the uptake of VMMC. Stakeholder engagement entails the involvement of those who have a role in, are interested in and are affected by the activities and goals of a programme (Ngomi, 2014). This implies that those who have a role in VMMC, are interested in and are affected by the activities and goals of the programme on VMMC in the community are consulted before the inception of the programme. Lissouba et al. (2010), Male Circumcision Consortium (2010), Lanham, L’Engle, Loolpapit, and Oguma (2012) as well as Katisi and Daniel (2015) reported that stakeholder engagement at all levels of the implementation process of a health programme enhances its success. This study, therefore, recommends government and other providers of VMMC to engage of all important stakeholders such as men, women, and village elders to boost the uptake of VMMC among men in Botswana.

Other perceived barriers that came out of the study were the lack of women involvement in VMMC. Lanham et al. (2012) opined that since women play an important role in decision-making around male circumcision, especially when it comes to adult men who are married, specific interventions need to be developed to increase their awareness and generate support for their partners and sons. The investigators of this study also suggest that women should not be overlooked when it comes to policymaking and programming on VMMC.

The lack of traditional leadership's support as a key barrier of the uptake of VMMC and was reported in two qualitative studies conducted in Botswana by Sabone et al. (2013) and Katisi and Daniel (2015). Similarly, this study found that the lack of traditional leadership's support is a key barrier of the uptake of VMMC in Kweneng East, Botswana. However, other barriers of the uptake of VMMC such as gender and age, potential tension between medical science and traditional knowledge systems; infrastructural and system challenges such as long distances to treatment centres, long waiting periods, and shortage of staﬀ and equipment and the conflict between traditional male circumcision and VMMC reported by these studies were not found in this study. This could be as a result of the time difference in the conduct of these studies as the other two previous studies were conducted some years ago and therefore the changes in the social structures may play a key role in the uptake of VMMC.

This study revealed that cultural beliefs were perceived as a barrier of the uptake of VMMC. Participants indicated that it is a taboo for parents to discuss sexual matters with children. The lack of communication on sexual matters between parents and their children often denied the sharing of information whereby young people could learn more about VMMC and some misconceptions about VMMC could be dispelled. Therefore, this predisposition by the parents or guardians, as well as the taboo in the Bakwena culture to openly discuss sexual matters made it difficult for young people to voluntarily engage in VMMC initiatives. These findings are consistent with the results of a qualitative study conducted in Botswana which revealed that older men were not comfortable being addressed by youth on male circumcision (Sabone et al., 2013). This study, therefore, recommends the provision of gender-and-culture sensitive VMMC to remove the barriers of the uptake of VMMC among men in Botswana (Masese, Chimango, & Mbirimtengerenji, 2017).

In this study, religious beliefs on VMMC influenced the uptake of VMMC among men. The findings of this study showed that positive religious beliefs towards VMMC encouraged men to use VMMC whilst negative religious beliefs towards VMMC refrained men from using VMMC. These findings corroborate the results of a study conducted among a non-circumcising society in rural western Kenya reporting that most participants were attached to their religious beliefs and were directed by it (Obure, Nyambedha, & Oindo, 2011). Religion has a huge influence on people and can influence the reception to VMMC programmes (Sewankambo & Mafigiri, 2017). The findings imply that religious leaders and institutions could be used to promote VMMC programmes. The findings of this study have implications for policy, practice and research. Theory of culturally congruent care stipulates that clients’ culture, beliefs and value systems should be considered when developing and implementing health care interventions (Abitz, 2016; Albougami et al., 2016; Schim et al., 2007). Furthermore, the understanding of the local context and meanings of VMMC are a crucial aspect of the implementation of the VMMC interventions in Eastern and Southern Africa (Khumalo-Sakutukwa et al., 2013). Therefore, the provision of VMMC services should not be done haphazardly without considering the influence of the value systems on the uptake of VMMC. Donor agencies and governments stand the risk of wasting resources if cultural sensitivities and well-monitored implementation frameworks are not given priority. Ignoring the cultural gatekeepers of service provision, such as consulting the appropriate authorities and stakeholders using the right channels of communication, and provision of other VMMC-friendly services will lead to resistance and lack of compliance, which will result in failure to achieve the goal of reducing HIV infections. Also, women should be encouraged to support their partners and men, in general, to patronise male circumcision programmes by increasing their awareness when such programmes are in force and encouraging them to support their partners and sons.

The researchers in this study recommend the provision of culturally congruent VMMC services to enhance the success of VMMC interventions. Cultural competence is required among VMMC providers to enable them to provide effective services within the cultural context of individuals, families, and communities. Culturally competence means VMMC providers hold a deep respect for clients’ cultural differences and understand the effects of the interventions provided to the clients and their culture (Abitz, 2016). VMMC providers need to overcome cultural ignorance by incorporating transcultural health care in practice, so as to avoid cultural impositions. There is a need for the development of in-service training programmes for service providers on the practice of a culturally congruent VMMC model and guidelines. Policymakers need to develop culturally congruent VMMC models, frameworks and guidelines to promote the success of VMMC interventions.

This study had several limitations. As with most qualitative studies, the generalisability of findings to other settings is limited due to the small sample size used in this study. A purposeful sampling method used to select participants for this study does not ensure a representative sample of the target population. The study did not obtain the views of other important groups such women, traditional leaders, other male population groups such as those aged 10–17 years, 50–64 years and other tribes which could have provided a comprehensive understanding of the perceived factors influencing the uptake of VMMC among men in Botswana.

Despite these shortcomings, the study contributes to ongoing research on the factors influencing the uptake of VMMC among men in Botswana. The findings of this study indicate that value systems of stakeholder consultation in the community, cultural and religious beliefs had contributed to the slow uptake of VMMC by men in Kweneng East, Botswana. A systematic review of the barriers and facilitators of the uptake of VMMC in priority countries revealed that the key barriers were; VMMC being perceived as practised by other or foreign cultures or religions, fear of pain caused by the procedure, futility of VMMC because of low HIV risk behaviour and still needing to use condoms (Carrasco, Wilkinson, & Mah, 2016). The findings of this study have theoretical relevance to similar study settings. However, the findings should be interpreted with caution and within the context of Bakwena men in Kweneng East, Botswana.

This study suggests many areas for future research. Further studies should investigate the perceptions of other important groups such women, traditional leaders, other male age groups such as 10–17 years, 50–64 years and other tribes in order to obtain a comprehensive understanding of the factors influencing the uptake of VMMC among men in Botswana.

It is concluded that the perceived factors influencing the uptake of VMMC among men in Kweneng East, Botswana were value systems associated with stakeholder consultation in the community, cultural beliefs and religious beliefs, and these factors to a larger extent deterred men from using VMMC services. To boost the uptake of VMMC and provide culturally congruent VMMC services among men in Kweneng East, Botswana, the study recommends that government and other providers of VMMC should consider the influence of value systems on the uptake of VMMC.
